# Methicillin-resistant *Staphylococcus aureus* in cystic fibrosis patients: do we need to care? A cohort study

**DOI:** 10.1590/1516-3180.2016.0350240317

**Published:** 2017-08-21

**Authors:** Renata Wrobel Folescu Cohen, Tânia Wrobel Folescu, Pedro Daltro, Marcia Cristina Bastos Boechat, Danielle Ferreira Lima, Elizabeth Andrade Marques, Robson Souza Leão

**Affiliations:** I Assistant Professor of Pediatrics, School of Medical Sciences, Universidade do Estado do Rio de Janeiro (UERJ), and Pediatric Pulmonologist, Instituto Nacional de Saúde da Mulher da Criança e do Adolescente Fernandes Figueira, Fundação Instituto Oswaldo Cruz (IFF/Fiocruz), Rio de Janeiro (RJ), Brazil.; II Head of Pediatric Pulmonology, Instituto Nacional de Saúde da Mulher da Criança e do Adolescente Fernandes Figueira, Fundação Instituto Oswaldo Cruz (IFF/Fiocruz), Rio de Janeiro (RJ), Brazil.; III Radiologist, Instituto Nacional de Saúde da Mulher da Criança e do Adolescente Fernandes Figueira, Fundação Instituto Oswaldo Cruz (IFF/Fiocruz), Rio de Janeiro (RJ), Brazil.; IV Head of Radiology, Instituto Nacional de Saúde da Mulher da Criança e do Adolescente Fernandes Figueira, Fundação Instituto Oswaldo Cruz (IFF/Fiocruz), Rio de Janeiro (RJ), Brazil.; V Fellow, Department of Microbiology, Immunology and Parasitology, School of Medical Sciences, Universidade do Estado do Rio de Janeiro (UERJ), Rio de Janeiro (RJ), Brazil.; VI Professor, Department of Microbiology, Immunology and Parasitology, School of Medical Sciences, Universidade do Estado do Rio de Janeiro (UERJ), Rio de Janeiro (RJ), Brazil.

**Keywords:** Cystic fibrosis, Methicillin-resistant Staphylococcus aureus, Tomography, Spirometry, Body mass index

## Abstract

**CONTEXT AND OBJECTIVE:**

The prevalence of a variety of potentially pathogenic microorganisms in cystic fibrosis patients, such as methicillin-resistant *Staphylococcus aureus* (MRSA), has increased over the past decade. Given the increasing prevalence of MRSA and the few data available in the literature, better understanding of the clinical repercussions of colonization by this bacterium in cystic fibrosis patients becomes essential. This study aimed to evaluate the repercussions of chronic colonization by MRSA in cystic fibrosis patients.

**DESIGN AND SETTING:**

Retrospective cohort study from January 2004 to December 2013 in a cystic fibrosis reference center.

**METHODS:**

Each patient with cystic fibrosis was evaluated for nutritional status (body mass index, BMI, and BMI percentile), pulmonary function and tomographic abnormalities (modified Bhalla scores) at the time of chronic colonization by MRSA or methicillin-susceptible *Staphylococcus aureus* (MSSA) and throughout the study period.

**RESULTS:**

Twenty pairs of patients were included. There were no significant differences between the groups regarding nutritional characteristics. Spirometric data showed a trend towards greater obstruction of the airways in patients with MRSA. Patients with MRSA presented greater structural damage to their lungs, demonstrated not only by the total Bhalla score but also by its parameters individually.

**CONCLUSIONS:**

Patients colonized by MRSA presented greater functional and structural respiratory impairment at the time of chronic colonization. Disease progression was also faster in patients chronically colonized by MRSA than in those with MSSA. This was shown through comparisons that avoided possible confounding variables.

## INTRODUCTION

Bacterial respiratory infection starts early in cystic fibrosis (CF) patients and *Staphylococcus aureus* (*S. aureus*) and *Haemophilus influenzae* are the main pathogens in young patients.^1^ Increasing life expectancy has led to higher prevalence of new pathogens in these patients,^2^ especially methicillin-resistant *S. aureus* (MRSA).^3^ The prevalence of MRSA infection in CF patients in the United States rose from 2% in 2001 to 26.5% in 2014.^4^ While *Pseudomonas aeruginosa* and the *Burkholderia cepacia* complex are classically associated with respiratory deterioration and worsening of life expectancy, the clinical impact of MRSA colonization is not so clear.^5^

Since the clinical course of CF respiratory disease varies between patients, information that clearly depicts the conditions of the respiratory system and progression of pulmonary structural lesions is needed, especially among patients infected by MRSA. Nutritional status directly influences multisystemic involvement among CF patients and it has been considered that body mass index (BMI) is the most important parameter to be monitored. The association of better nutritional status with improved lung function is well documented and poor nutritional status with lower BMI is a risk factor for accelerated decline in lung function.^6^

Premature deaths continue to result directly or indirectly from loss of lung function. Therefore, spirometric measurements are important surrogate measures of disease progression, particularly forced expiratory volume in the first second (FEV_1_%) and predicted forced vital capacity (FVC%).^6^

High-resolution computed tomography (HRCT) of the chest is a specific imaging method that can be used to evaluate early airway disease and parenchymal lesions. Furthermore, it has high sensitivity and specificity for diagnosing lung injury and also allows better qualitative description of respiratory impairment.^7^^,^^8^ HRCT results are presented as graphical images, which may be converted to score values that are useful for evaluating disease severity. Since the first proposal for creation of a computed tomography scoring system to quantify structural abnormalities in CF patients,^9^ other scoring systems have been described and been shown to be reproducible and comparable.^7^ The modified Bhalla score^10^ was shown to be reproducible and reliable, regardless of the severity of the lesions, which suggests that it is applicable in clinical practice for CF cases.^11^

## OBJECTIVE

The aim of this study was to evaluate the clinical and tomographic impact of chronic MRSA infection in CF patients and compare these data with those from a group of individuals chronically colonized by methicillin-susceptible *S. aureus* (MSSA).

## METHODS

This retrospective cohort study was conducted in the main CF center for children and adolescents in Rio de Janeiro, Brazil: the Fernandes Figueira National Institute for Women’s, Children’s and Adolescents’ Health (Instituto Nacional de Saúde da Mulher, da Criança e do Adolescente Fernandes Figueira, Oswaldo Cruz Foundation, Ministry of Health), from January 2004 to December 2013. Approval was obtained from this institution’s ethics committee (number CAAE 27902814.9.0000.5269).

In this institution, respiratory secretions (either sputum or throat swabs in the case of children under two years of age who were unable to provide sputum samples) were cultured in accordance with standardized protocols that had been established for CF patients. This was done on a three-monthly basis throughout the study.^12^

The inclusion criteria for the patients were that they needed to have a CF diagnosis in accordance with the Cystic Fibrosis Foundation consensus;^13^^,^^14^ undergo regular clinical and laboratory follow-up during the study period; and present chronic colonization with *S. aureus* (MSSA or MRSA) in respiratory secretions (three or more isolations over a 12-month period).^15^^,^^16^ All patients that met eligibility criteria were consecutively included in the study and their data was recovered from medical records. Subjects who presented no respiratory colonization or were colonized with *B. cepacia* complex or presented intermittent colonization with *S. aureus* (less than three isolations over a 12-month period) were excluded from the study. No anti-staphylococcal prophylaxis is regularly used at this center. After the first isolation of MRSA (i.e. before chronic colonization), the patients underwent a course of antibiotics that included two weeks of rifampicin and trimethoprim-sulfamethoxazole and five days of topical mupirocin.

Each of the 20 chronic MRSA patients was matched with a control (chronic MSSA CF patient) in accordance with the following criteria: gender, age, time of chronic colonization with *S. aureus* (± 1 year) and chronic coinfection with *P. aeruginosa*.^17^

For each patient, medical record data were evaluated, including gender, age and pancreatic insufficiency (fecal fat or fecal elastase or need for exogenous replacement enzymes). The clinical outcome of each patient was also evaluated, considering the following parameters: BMI and BMI percentile for children over the age of two years, spirometric parameters such as predicted forced expiratory volume in the first minute (FEV_1_%) and predicted forced vital capacity (FVC%), and modified Bhalla scores for HRCT. These parameters were evaluated and compared, at the time of chronic colonization with MRSA and MSSA, and throughout the study period.

Nutritional status was monitored through BMI obtained at the time of each evaluation at the CF center. The best annual BMI results were selected. These were used for BMI percentile calculations (for patients 2-19 years old) through the tool available at http://www.cdc.gov/healthyweight/assessing/bmi.^18^

Lung function data were evaluated through retrospective analysis on spirometric reports. The spirometric examinations were performed using the Collins Survey II computerized system (Warren E. Collins Inc., Massachusetts, USA), on patients over the age of six years who had the cognitive ability to undergo the test. The absolute values were converted to percentages of predicted values, using reference equations from Knudson et al.^19^ The highest FEV_1_% and FVC% values reported for each patient every year were analyzed.

In this CF center, HRCT examinations were performed every two to four years when patients were clinically stable. These images were obtained using the ProSpeed-S™ device (General Electric, Milwaukee, WI, USA), with 1 mm slices every 10 mm, at 80 to 100 mAs (milliampere/second) and 120 kV (kilovolt), in windows of -1500 HU and at a level of 700 HU, without sedation, and with inspiration and expiration series. HRCT images were retrospectively analyzed by a pediatric radiologist and by a pulmonologist with more than 10 years of experience of CF, who had no information about any patient data. After meeting and discussing the classification adopted for the modified Bhalla score, both professionals applied it to each exam in a completely random order. The modified Bhalla score had been previously validated at another study at this institution.^11^ The total score is obtained from the sum of the values for the severity and/or extent of each morphological abnormality and can range from 0 (no abnormality) to 37 (severe abnormalities in all items).^10^

### Statistical analysis

Descriptive statistical analyses were performed through construction of tables and graphs and through summary measurements appropriate for each variable.

In order to study the trends of several variables over time, the annual rate of change was calculated by means of simple linear regression for each patient. The Wilcoxon nonparametric test was used to determine the statistical significance of differences between the MRSA and MSSA groups and between two moments. The Statistical Package for the Social Sciences (SPSS 17.0 for Windows) was used.

## RESULTS

Out of 170 CF patients who were followed up at this CF center, 20 (11.76%) fulfilled the criteria for chronic MRSA colonization. The comparison group was selected from among 70 CF patients who presented chronic colonization with MSSA. Twenty pairs of patients were included: 40% were female (8 pairs); 95% had pancreatic insufficiency (19 pairs); and 25% had *P. aeruginosa* coinfection (5 pairs).

The mean age at the time when cystic fibrosis was diagnosed was 2.5 years in the MRSA group and 2.1 years in the MSSA group. Seven patients were diagnosed through newborn screening test and the others through clinical features compatible with cystic fibrosis, with confirmation using sweat test. The mean follow-up time at the CF center was similar in the two groups (6.7 years for MRSA versus 7 years for MSSA). Other sociodemographic data are available as supplementary material (Annex).

In relation to nutritional status, the mean BMI values at the time of chronic colonization were similar: 15.5 (standard deviation, SD ± 2.2) in the MRSA group and 15.6 (SD ± 3.6) in the MSSA group, without significant statistical difference (P-value 0.823). The same trend was found for the mean BMI annual rate of change: 0.22 (SD ± 0.49) in the MRSA group and 0.19 (SD ± 0.44) in the MSSA group (P-value 0.852). Correspondingly, there was no statistically significant difference in BMI percentile between the time of chronic colonization (39.6 (SD ± 32.3) in MRSA; 39.8 in MSSA (SD ± 33.1); P-value 0.823) and thereafter: the mean annual rate of change in BMI percentile was 2 (SD ± 8) in MRSA and 1.1 (SD ± 6) in MSSA (P-value 0.852).

Spirometric data were available for 10 pairs of patients (who were older than six years of age). The mean values for FEV_1_% and FVC% were lower in the MRSA group at the time of chronic colonization. After chronic colonization, the mean annual rate of decline in FEV_1_% for the MRSA group was 3.2% (SD ± 1.8), while in MSSA it was 2.3% (SD ± 1.8), but there was no statistically significant difference between the groups (P-value 0.374). Meanwhile, the mean annual rate of decline in FVC% was also higher for the MRSA group (3.2% per year, SD ± 1.9) than for the MSSA group (1.8% per year (SD ± 1.7), with a statistically significant difference (P-value 0.038) (Table 1).

**Table 1.**
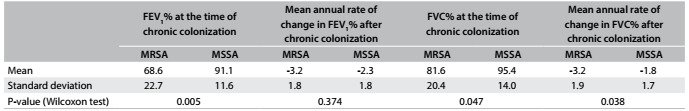
Spirometric data from patients with methicillin-resistant *Staphylococcus aureus* (MRSA) and methicillin-susceptible *Staphylococcus aureus* (MSSA): at the time of chronic colonization and mean annual rate of change after chronic colonization (n = 10 pairs)

FEV_1_% = predicted percentage forced expiratory volume in first second; FVC% = predicted percentage forced vital capacity.

The modified Bhalla score results from HRCT at the time of chronic colonization are described in Table 2. Evaluation of each pair at the time of chronic colonization showed that the Bhalla scores were higher in 16 patients with MRSA. At this time, the mean Bhalla score in the MRSA group was twice the score in the MSSA group (6.5 and 3.3, respectively), with a statistically significant difference (P-value 0.002) (Table 2). The annual rate of change of the Bhalla score was higher in 19 patients with MRSA, thus showing that there was faster progression of lung injury in the MRSA group. The mean annual rate of change in Bhalla score was four times higher in the MRSA group (1.7 points per year in MRSA and 0.4 points per year in MSSA), with a statistically significant difference (P-value < 0.001) (Table 2).

**Table 2.**
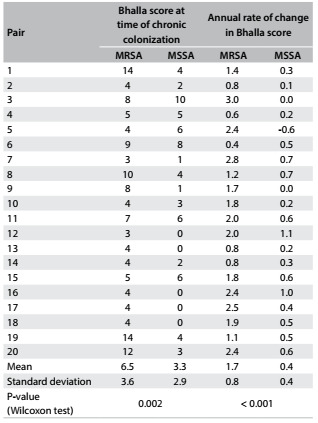
Bhalla score among patients with methicillin-resistant *Staphylococcus aureus* (MRSA) and methicillin-susceptible *Staphylococcus aureus* (MSSA): at the time of chronic colonization and mean annual rate of change after chronic colonization (n = 20 pairs)

Regarding the scores for each Bhalla parameter at the time of chronic colonization, only the findings of bronchiectasis and mucous plugging were more severe in the MRSA group. There were no statistically significant differences between the groups for the other parameters (Table 3). However, the HRCT performed after chronic colonization (mean time elapsed of 4.5 years) showed that all the Bhalla scores were significantly worse in the MRSA group (Table 4). At this time, all the MRSA patients presented air trapping, mosaic attenuation/perfusion pattern, bronchial wall thickening and mucous plugging. Bronchiectasis was found in 90% of the MRSA patients.

**Table 3.**
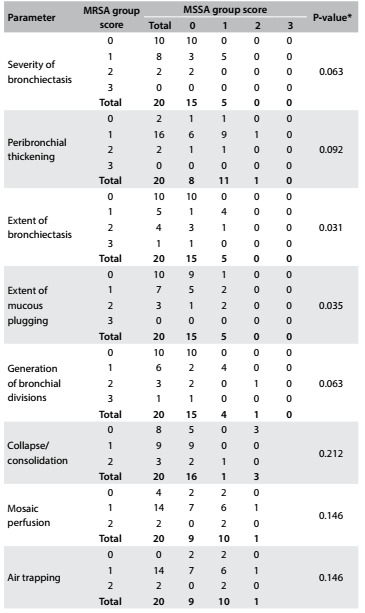
Number of patients with each score for the different parameters at the time of chronic colonization in the methicillin-resistant *Staphylococcus aureus* (MRSA) and methicillin-susceptible *Staphylococcus aureus* (MSSA) groups (n = 20 pairs)

*P-value obtained via Wilcoxon test. Note: The items “sacculations/abscesses”; “emphysema”; “acinar nodule”; “thickening of intralobular septa” and “ground glass” were not analyzed individually because almost all scores were zero.

**Table 4.**
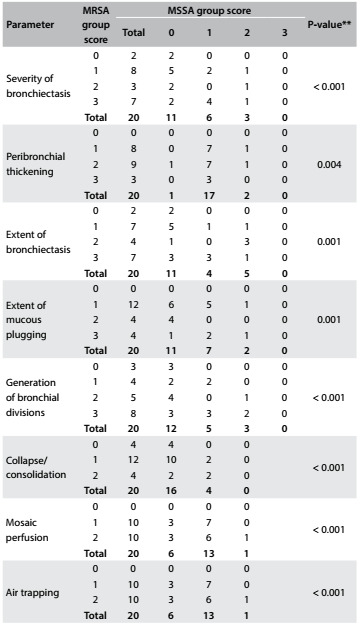
Number of patients with each score for the different parameters after* chronic colonization in the methicillin-resistant *Staphylococcus aureus* (MRSA) and methicillin-susceptible *Staphylococcus aureus* (MSSA) groups (n = 20 pairs)

*Mean time elapsed of 4.5 years; **P-value obtained via Wilcoxon test. Note: The items “sacculations/abscesses”; “emphysema”; “acinar nodule”; “thickening of intralobular septa” and “ground glass” were not analyzed individually because almost all scores were zero.

## DISCUSSION

In the present study, careful pairing was done, considering age, gender and chronic colonization with *P. aeruginosa,* in order to eliminate confounding factors and effectively measure the impact of MRSA colonization in cystic fibrosis. It is known that there is a female survival disadvantage in CF cases^20^ and that chronic *P. aeruginosa* infection results in a prolonged inflammatory response. The latter is believed to cause respiratory tissue injury, which leads to progressive loss of lung function and adversely affects survival.^21^

Despite increasing prevalence of and morbidity associated with MRSA colonization in CF patients worldwide, little is known so far about its effects on lung damage. It is not clear whether MRSA colonization is simply a marker of greater severity of lung disease^5^ or is an independent contributor towards lung function decline.^15^ In our study, the lung function data (FEV_1_% and FVC%) were worse at the time of chronic MRSA colonization. From then on, the annual decline in FEV_1_% was 39% higher in the MRSA group. These findings showed a trend towards worse outcomes, although without statistical significance, probably due to the sample size. They highlight the impact of chronic MRSA colonization on airflow obstruction in CF patients, compared with those who only had MSSA. These results are consistent with those from other studies in which MRSA was shown to have a negative impact on lung function.^15^^,^^22^ In a 10-year longitudinal cohort study that included 17,357 patients in the United States, it was reported that the decline in lung function was faster among patients with chronic MRSA colonization, after adjustment for possible confounding variables. In that cohort, the decline in predicted FEV_1_ of 2.06%/year was 43% more rapid than the 1.44% predicted/year among those without MRSA (P-value < 0.001).^15^ Ren et al., using a large database of CF patients, showed that out of 1834 patients presenting *S. aureus* in respiratory tract cultures, those who were less than 18 years of age and had MRSA showed significantly greater airflow obstruction (FEV_1_), compared with MSSA patients.^22^ However, their study did not match patients according to age and gender, which are significant confounders and could have influenced the outcome. Despite the knowledge that the rate of transient infection with MRSA can reach up to 69%,^15^ patients with only one positive culture could still be included in the MRSA group in their study.^22^ Our study provides consistent data, in that it only included patients with chronic MRSA colonization, so as to provide better understanding of the lung function repercussions of this bacterium.

Lung function data (especially FEV_1_%) and the respiratory exacerbation rate have been widely used as sensitive markers. However, since advances in CF treatment have been able to delay the loss of lung function, other measurements have been revealed to be important for monitoring CF patients, including nutritional indices such as BMI.^6^ The association of better nutritional status with improved lung function is well documented, and poor nutrition is a risk factor for accelerated decline in lung function. Data from 6,835 CF patients in the German CF Registry (1995-2005) found that low BMI (< 19 kg/m^2^) and low FEV_1_ (< 80%) correlated with mortality.^6^ Identifying risk factors that may contribute to the rate of decline of lung function and BMI may help in focusing interventions.^6^ Few studies have discussed the effects of MRSA colonization on nutritional status. A retrospective study (2003-2007) identified 12 pediatric patients presenting MRSA colonization. After one year of MRSA colonization, only one patient showed a decrease in BMI percentile.^23^ In our study, there were no significant differences between the groups regarding BMI or BMI percentile at the time of chronic MRSA colonization, or in the annual rate of decline of BMI.

With the recent advances in therapeutic approaches towards CF, it is essential to identify tools that can monitor lung disease progression and response to treatment.^7^ Imaging examinations are able to detect early disease progression, thereby contributing to treatment effectiveness and quality of life among CF patients. Furthermore, HRCT can be used both for patients who are unable to undergo spirometry and for analysis on the two tests, since HRCT shows early changes in patients with normal spirometry.^24^ Several studies have shown that despite stable spirometric parameters, HRCT scores show annual progression in total CT score.^7^ Despite the well-established importance of HRCT scores for CF, few studies have made reference to these scores in different microbiological groups,^11^ with no published data regarding MRSA colonized patients. In this context, our study highlights the importance of HRCT scores in CF patients with chronic colonization with MRSA, thus making a significant contribution towards aiding clinicians who deal with this group of patients.

The importance of certain lung morphological abnormalities is emphasized in the modified Bhalla scoring system. The parameter “mucous plugs”, for example, is scored separately because of their crucial role in the pathogenesis of bronchiectasis. Likewise, peribronchial thickening reflects the presence of recurrent chronic infection that results in bronchial and peribronchial inflammation and/or fibrosis, which should be scored separately. These two abnormalities are of great importance in managing CF, since they might suggest the need for a specific therapeutic approach.^9^ Simultaneously, presence of bronchiectasis in CF cases has been correlated with chronic bronchial disease, consequent to persistent inflammation and subsequent weakening of the airway wall, thereby resulting in irreversible distension of the bronchial walls.^25^

The importance of these considerations stood out in the present study, from which it was evident that abnormalities such as bronchiectasis, peribronchial thickening and mucous plug formation were present in almost all patients with chronic MRSA colonization. These results are concordant with analyses from other studies in which the three most prevalent abnormalities were bronchiectasis (86-90%), peribronchial thickening (53-80%) and mucous plugging (63-79%).^11^^,^^26^^,^^27^^,^^28^

In the present study, the modified Bhalla score values demonstrated that MRSA patients showed greater structural lung damage at the time of chronic colonization. At that time, detailed analysis on each parameter of the Bhalla score showed that for two of them, there were statistically significant differences between the groups (extent of bronchiectasis and mucous plugging). After chronic MRSA colonization, there was marked progression of structural lung damage, as demonstrated through the total modified Bhalla score and all its parameters individually. The presence of bronchiectasis in all patients with chronic MRSA colonization highlights the severity of injuries in this group and confirms the association of MRSA colonization with progression of structural lung injury in CF patients.

Although the other more prevalent injuries, such as peribronchial thickening, mucous plugging and mosaic perfusion, are known to be reversible, these were also more frequent in the MRSA group. This evidence supports the hypothesis that chronic MRSA colonization leads to a more prominent inflammatory response and earlier structural lesions. The mechanisms proposed for this effect may be similar to those proposed for MRSA pneumonia in non-CF patients. It is possible that MRSA remains in the respiratory tract for longer times because of its resistance to commonly used antibiotics. Moreover, presence of toxins and virulence factors may mediate greater degrees of airway inflammation.^29^^,^^30^

The present study is unprecedented in Brazil. It determined the relationship between clinical data and chronic colonization with MRSA, an emergent bacterium in CF cases for which greater understanding of the impact of chronic infection is needed. However, the present study has limitations because of its retrospective nature and small sample size, especially in relation to lung function tests, and these may have interfered with the statistical significance of the results. Despite these limitations, the implications of our findings strengthen the argument for development of possible evidence-based MRSA eradication measures and serve as a starting point for future studies on CF patients with chronic MRSA colonization.

## CONCLUSION

These MRSA-colonized CF patients presented greater degrees of functional and structural lung disease at the time of chronic colonization, and disease progression seemed to be faster. This was shown through comparisons in which potential confounding variables were controlled for.
